# Contribution of 15 Years (2007–2022) of Indo-US Training Partnerships to the Emergency Physician Workforce Capacity in India

**DOI:** 10.5811/westjem.59912

**Published:** 2023-07-17

**Authors:** Joseph D. Ciano, Katherine Douglass, Kevin J. Davey, Shweta Gidwani, Ankur Verma, Sanjay Jaiswal, John Acerra

**Affiliations:** * Northwell Long Island Jewish Medical Center, Department of Emergency Medicine, Glen Oaks, New York; † George Washington University School of Medicine & Health Sciences, Department of Emergency Medicine, Washington DC; ‡ Chelsea & Westminster NHS Trust, London, United Kingdom; § Max Super Specialty Hospital, Patparganj, Department of Emergency Medicine, New Delhi, India

## Abstract

**Background:**

Indo-US Masters in Emergency Medicine (MEM) certification courses are rigorous three-year emergency medicine (EM) training courses that operate as a partnership between affiliate hospitals or universities in the United States with established EM training programs and local partner sites in India. Throughout their 15 years of operation, these global training partnerships have contributed to the EM workforce in India. Our objective in this study was to describe Indo-US MEM program graduates, their work environments, and their contribution to the growth of academic EM and to the coronavirus disease 2019 (COVID-19) response.

**Methods:**

An electronic survey was created by US and Indian MEM course stakeholders and distributed to 714 US-affiliated MEM program graduates. The survey questions investigated where graduates were working, their work environments and involvement in teaching and research, and their involvement in the COVID-19 response. We consolidated the results into three domains: work environment and clinical contribution; academic contribution; and contribution to the COVID-19 response.

**Results:**

The survey response rate was 46.9% (335 responses). Most graduates reported working within India (210, 62.7%) and in an emergency department (ED) setting (304, 91.0%). The most common reason for practicing outside of India was difficulty with formal MEM certificate recognition within India (97, 79.5%). Over half of graduates reported dedicating over 25% of their work hours to teaching others about EM (223, 66.6%), about half reported presenting research projects at conferences on the regional, national, or international level (168, 50.5%), and almost all graduates were engaged in treating COVID-19 patients during the pandemic (333, 99.4%). Most graduates agreed or strongly agreed that they were satisfied with their overall MEM training (296, 88.4%) and confident in their ability to practice EM (306, 91.6%).

**Conclusion:**

Indo-US MEM graduates have made a notable contribution to EM in India through clinical service delivery, teaching, and research, even more essential in the context of the COVID-19 pandemic. The roles of these graduates should be acknowledged and can contribute further to expand EM specialty and systems development across India.

## INTRODUCTION

Emergency medicine (EM) in India has reached many developmental milestones since its formal recognition in 2009 by the Medical Council of India. Postgraduate EM training programs have grown in number in recent years; however, the number of available training positions remains vastly inadequate for a country of 1.32 billion people.^1^,^2^ Road traffic injuries and non-communicable diseases, such as ischemic heart disease and stroke, are amongst the top causes of morbidity and mortality across India with a growing prevalence across all states.^1^,^2^ These conditions require time-sensitive interventions and diagnostics that emergency physicians (EP) are trained to recognize and provide.

Accreditation of EM training programs in India is overseen by the National Medical Commission (NMC, formerly the Medical Council of India) and the National Board of Examinations (NBE).^3^–^6^ As of 2018, the Medical Council of India had fully approved only 73 training positions for EM within government-funded public hospitals, offered in 29 of 460 medical colleges in India covering just eight states and two union territories. As of March 2022, these had increased to 187 training positions.^4^ In 2014 the NBE, which oversees residency training in private hospitals, also recognized EM as a specialty and set out its EM curriculum and examination process. By 2018, the NBE was offering an additional 121 training positions, although 52 of these remained vacant as of July 2017.^7^

Even if all the training positions were filled, India has the capacity to train only 308 EPs annually. By comparison, the United States has 2,278 EM training seats per year, and 58,000 active board-certified EPs for a population less than one quarter the size of that of India.^8^,^9^ The United Kingdom’s (UK) publicly funded National Health Service has one EP for every 10,000 people. India would need about 132,000 EPs *today* to reach similar doctor-population ratios.^10^ This gap in clinician ratios has been felt by hospitals keen to provide emergency and urgent care services in India. In response to the EM training gap, a wide array of EM educational programs, courses, and conferences have been developed in India over the last two decades.^3^,^10^

Similar partnerships to develop EM exist in other countries in the early stages of specialty development.^11^ US-MEM programs operate as a partnership between affiliate hospitals or universities in the US that have established EM training programs and local partner sites in India.^10^–^13^ The MEM programs are rigorous three-year programs that emulate the structure of US training programs, including supervised clinical experience in EM, clinical rotations throughout relevant specialties, didactics, trainee assessment, and examinations to measure competence.^14^ Similar to many Western residency training programs, most Indo-US MEM courses follow a 36-month modular curriculum with required readings from EM texts (eg, *Tintinalli’s Emergency Medicine*) and assigned questions from purchased question banks.^15^

**Population Health Research Capsule**

What do we already know about this issue?

*Emergency medicine training positions in India have increased but remain insufficient. Indo-US Masters in EM (MEM) programs build emergency physician capacity in India*.

What was the research question?

*How have Indo-US MEM graduates contributed to physician workforce capacity in India over the last 15 years?*

What was the major finding of the study?

*Most MEM graduates work within India (210, 62.7%) in an ED setting (304, 91.0%)*.

How does this improve population health?

*Developing emergency physician workforce capacity will contribute to improved population health through clinical service delivery and EM specialty development*.

Institutional faculty affiliates from the US visit India throughout the year to assist local faculty in bedside teaching, didactics, resident assessments, and exams.^15^ Most course sites offer residents the opportunity to participate in a rotation at the partnering US institution, although most residents complete their MEM course entirely in India. Since the inception of the first MEM program in 2007, MEM programs have trained over 700 graduates throughout India. Given the duration of operation of these training programs, we sought to describe the contributions of Indo-US MEM graduates as part of the postgraduate EM training landscape in India.

We believe that understanding these Indo-US models is of particular importance and relevance now, both because of the vast, unmet clinician gap and the evolving political and regulatory landscape in India, which is edging towards more international university-partnership models of education and training. Of note, on 5 January 2023, the University Grants Commission unveiled a draft seeking public feedback on the proposal to facilitate entry and operation of foreign universities in India.^16^ In this paper, we focus on describing details of 15 years (2007–2022) of Indo-US training partnerships and their impact on EP workforce capacity in India with respect to the graduates’ work environments and their contribution to the growth of academic EM and more recent contribution to the coronavirus 2019 (COVID-19) response.

## METHODS

An electronic survey was created and distributed to 714 US-affiliated Indian MEM program graduates. Survey questions were written by US institutional stakeholders involved in Indian MEM program development and operations, namely Northwell Health in New York and George Washington University (GW) in Washington, DC. Key program faculty members in India, all of whom are MEM alumni, were asked to review the survey questions and provide feedback during the survey development phase. Some changes were made based on these recommendations prior to its distribution. The survey was written in English, as a working proficiency in English is a requirement of MEM course admission. No identifying information was collected, and the project was deemed exempt by the Northwell Health Institutional Review Board.

The survey consisted of 32–40 items, dependent on the use of branching logic to create a flow of relevant questions. Response options were a combination of multiple-choice; a “check all that apply” click box; short-answer questions with free text; and questions with a five-point Likert scale. The purpose of the questions was to determine where graduates were working (inside or outside India), their work environments (clinical, non-clinical, research, within an emergency department [ED] vs another setting, etc), involvement in teaching and research, confidence in their EM practice, satisfaction with their MEM training, and involvement in the COVID-19 response. The survey instrument is included in Appendix A.

Survey recipients included only MEM programs affiliated with US institutions. Program directors were contacted to collect contact information for program graduates. The MEM training sites that we contacted include those that work with the following affiliates in the US: GW; Northwell Health; State University of New York (SUNY) Upstate (Syracuse, NY); and the University of Maryland (UMD) (Baltimore, MD). At the time of our project, the total number of MEM graduates for each site was as follows: 647 (GW); 46 (Northwell Health); 13 (SUNY Upstate); and 20 (UMD). Of the 726 MEM graduates, contact information was obtained for 714 graduates (98.3%). Surveys were distributed via email to all 714 MEM program graduates from December 2021–February 2022 with 2–3 reminder emails sent to increase response rates. Survey results were collected electronically with REDCap research software hosted at Northwell Health and consolidated into domains for analysis. REDCap (Research Electronic Data Capture) is a secure, web-based software platform designed to support data capture for research studies.

## RESULTS

The total survey response rate was 46.9% (335 responses). The survey response rate for each institutional affiliate was as follows: GW, 46% (300 responses); Northwell Health 70% (32 responses); SUNY Upstate 15% (two responses); and UMD, 5% (one response). Results were categorized into three major domains. These domains included the clinical contribution to EM, academic contribution to EM, and involvement in the COVID-19 response. Key results are described below with more detailed results depicted in Tables 1–5. The responses to each question are expressed in the tables in order of decreasing prevalence. Demographic information is summarized in Table 1.

The majority of MEM graduates reported working within India (210, 62.7%) and within an ED work environment (304, 91.0%). The UK (54, 19.4%) and United Arab Emirates (17, 5.1%) were the most common locations for work outside India. The most common reason for choosing to practice outside India was due to difficulty with formal recognition of the MEM certificate within India (97, 79.5%), followed by differences in salary (71, 58.2%) and standard of living (58, 47.5%). Most graduates practicing outside India reported the decision to leave India was made after completing the MEM program (96, 80.0%). Graduates reported working mainly in the Delhi National Capital Region (NCR) (35, 16.8%), Maharashtra (28, 13.5%), and Kerala (31, 14.9%); however, graduates’ practice locations were broadly located throughout India.

More than half of graduates reported dedicating over 25% of their work hours to teaching others about EM, with nurses (218, 66.5%), other physicians (191, 58.2%), paramedics (191, 58.2%), and community members (129, 39.3%) listed as common recipients of educational activities. About half of all graduates reported presenting research projects at academic conferences (168, 50.5%) and endorsed membership in national EM professional organizations, such as the Society for Emergency Medicine India (SEMI) and INDUS-EM (170, 50.7%). Almost all graduates were engaged in treating COVID-19 patients during the pandemic (333, 99.4%) with more than 70% of graduates dedicating over half of their work hours to COVID-19 patient care. Most graduates agreed or strongly agreed that they were satisfied with their overall MEM training (296, 88.4%), well prepared to work in the ED (311, 92.8%), and confident in their ability to practice EM (306, 91.6%).

## DISCUSSION

Data from the current study describes the contribution of Indo-US MEM graduates to EM specialty development in India over the last 15 years.

### Work Environment and Clinical Contribution

The majority of graduates reported that they continue to practice within India (62.7%) and in an ED setting (91%). Of those that did leave, the main reasons cited include lack of recognition of their MEM certificate in India (79.5%), better salaries (58.2%), and greater opportunities for professional growth (56.6%). Since their inception in 2007, Indo-US MEM courses have trained over 700 EPs, and a majority are still working in EDs in India. The number of postgraduate Diplomate of National Board (DNB) and MD seats offered in EM are still far fewer than those offered in other countries, such as the US or UK, despite India having a substantially larger population.^5^,^13^,^17^ The MEM seats may continue to fill the gap in India’s EM clinical workforce until DNB and MD seats in EM have significantly increased. Graduates of MEM programs working in India should be looked at as a resource for specialty development in India. Many serve in leadership roles in their hospitals and take part in educating medical students and future EPs.

Steps should be taken to recognize MEM graduates as EM specialists to improve retention of physicians who receive additional EM training after their MBBS [Bachelor of Medicine and Bachelor of Surgery, which is equivalent to MD degree in the US]. This may lead to more professional opportunities throughout the private and public sector, improved job security, and more competitive salaries.

### Geographical Distribution of Skilled Emergency Workforce

**Figure 1:**
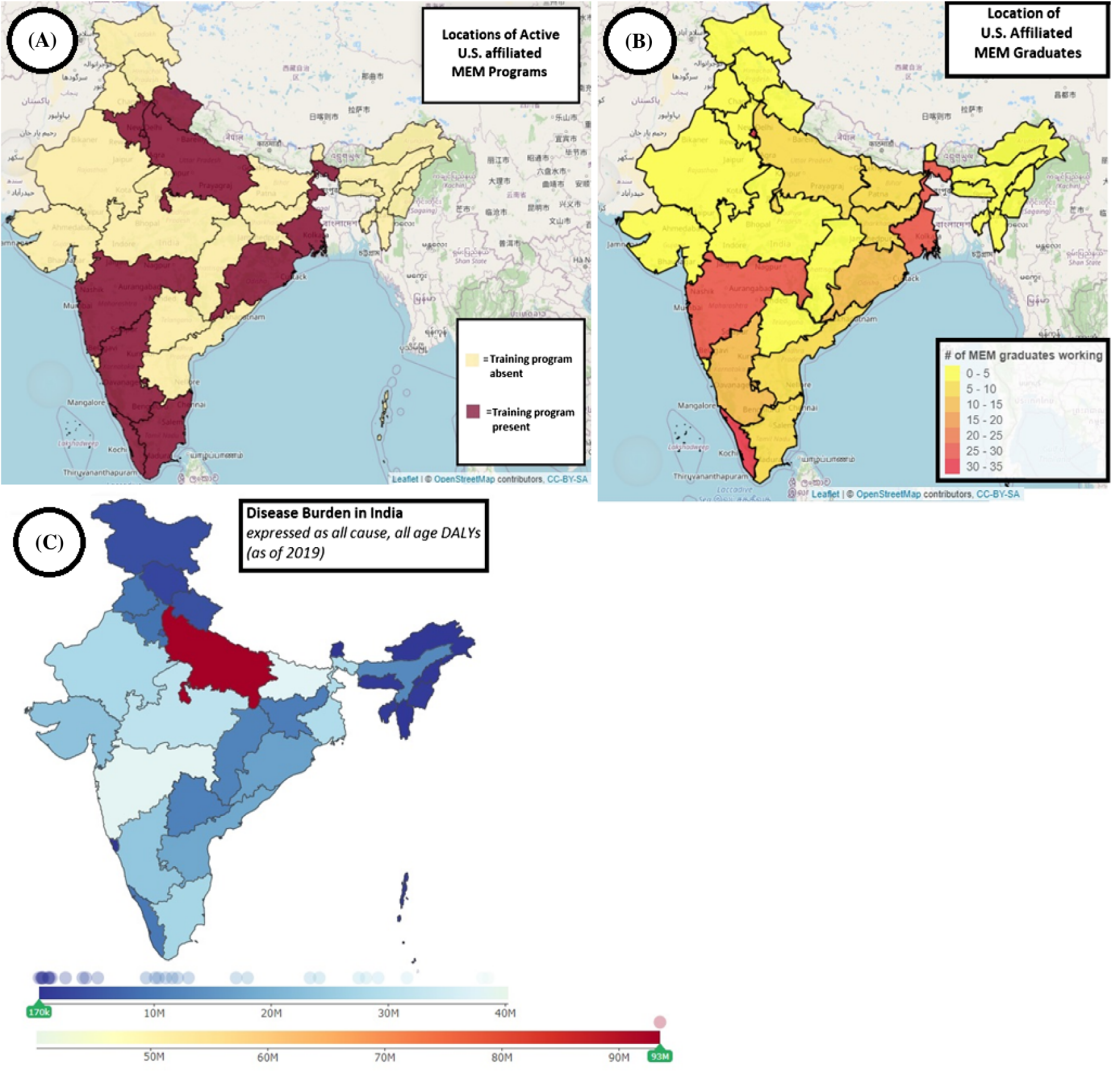
Geographical heat maps of India depicting the locations of active U. S. Masters in Emergency Medicine (US-MEM) (1A), the locations of US-MEM graduates (1B), and differences in disease burden as all-cause disability-adjusted life years (1C) depicted using the GBD India Compare tool from the Indian Council of Medical Research.

Our data indicates that many graduates choose to work in more well-resourced urban areas, such as Delhi NCR, Kerala, and Maharashtra, a common trend seen across different types of healthcare workers throughout India.^18^ These same states have doctor densities above the national average of 79.7 doctors per 100,000 population.^18^ While this is not surprising, it is also noteworthy that 10.6% of MEM graduates are practicing in lower-income-per-capita states, such as Uttar Pradesh (eight, 3.8%), Jharkhand (seven, 3.4%), Bihar (six, 2.9%), and Madhya Pradesh (one, 0.5%). These states have some of the lowest physician densities with Bihar state being one of the lowest at 52.6 physicians per 100,000 population.^9^ Lower-income-per-capita states in India have been transitioning, albeit at a slower rate than the wealthier states, towards a predominant non-communicable disease burden, similar to the patterns of disease seen around the world.^19^ Since 2000, lower income states have attributed more than 50% of their total deaths to non-communicable diseases.^1^ This is relevant to EM workforce distribution as the acute management of non-communicable disease complications, such as myocardial infarctions, strokes, and diabetic emergencies, is an area of EM expertise. Although MEM graduate numbers from the current study in low-income states are small, their contribution is notable in the context of low specialist-physician densities and poor funding. To highlight this concept, we created geographical heat maps of India describing the locations of active US-MEM programs (Figure 1A) and the locations of working US MEM graduates (Figure 1B) using a web-enabled heat geo-mapping tool.^20^ A heat map of India demonstrating differences in disease burden as all-cause disability-adjusted life years (DALYs) [years of life lost due to premature mortality] (Figure 1C) was created using the Global Burden of Disease India Compare tool from the Indian Council of Medical Research, Public Health Foundation of India, and Institute for Health Metrics and Evaluation.^21^

Locations of active training programs (Figure 1A) and states where graduates choose to work (Figure 1B) overlap with Indian states with mid-range to high disease burdens (Figure 1C) as expressed by states with light blue-white and maroon colors. Some examples in Figure 1C are Uttar Pradesh, Bihar, Maharashtra, and West Bengal. Although the high disease burden of these states may in part be due to these being some of the most populous states of India, the high DALYs of these locations cannot be denied. Many of these states also house US-affiliated MEM programs to promote the clinical and academic development of EM.

In addition to the need to grow EP training opportunities, emigration of skilled physicians from India is another barrier to creating and maintaining a robust workforce. We found that 37.3% of MEM graduates report practicing medicine outside India after graduation. High-income countries, such as the UK, Canada, the US, Gulf countries, and Australia, have historically functioned as common emigration sites for trained medical specialists from India.^22^,^23^ The Organization for Economic Cooperation and Development estimated that over 90,000 physicians who trained in India now work outside India with the US and UK as the most common receiving countries.^22^ This migration of MEM graduates is not unique to EM, to this partnership program, or to India. The net migration of healthcare workers from low- and middle-income countries to higher income countries is a cause for growing concern globally, resulting in a few articles on a more managed form of circular migration.^24^,^25^ This consideration could prompt action in higher recognition of MEM training and the need for more opportunities for professional growth.

### Academic Contributions

Many graduates report involvement in academic teaching and research activities. While more than half of graduates cite involvement in the education and training of other physicians, nurses, paramedics, laypeople, and ayurvedic healers are also taught by MEM graduates. This illustrates the broad reach across disciplines and fields by which graduate-led education can occur. It also highlights an important concept in global EM capacity-building as the training of one EP can impact the knowledge and experience of multiple other members of the healthcare team and surrounding community. Graduates imparting their EM knowledge to others reaches far beyond their contribution to the country’s specialized clinical workforce; it expands the competency and specialized clinical knowledge of the hospital-based healthcare team, nurses, prehospital personnel, and community members. Education may also empower others to continue to grow their knowledge and participate in other EM-based initiatives and training.

Other academic pursuits reported by graduates include presenting research abstracts or projects at conferences, membership in national EM organizations, and publishing abstracts, textbook chapters, or research articles. Publishing research and educational materials creates concrete deliverables that can enhance the knowledge of others. Publications can be used to support context-specific EM policies, such as new emergency medical services protocols, and they can assist in local advocacy. Publication of scholarly work also connects India to a global academic community and establishes the role of MEM graduates as an emerging expert resource in the field of EM.

Academic EM organizations in India, such as SEMI, INDUS-EM, and the Academic College of Emergency Experts in India, should focus on expanding opportunities for professional growth. Offering more courses on professional development, expanding teaching and research skills, and increasing the number of educational conferences and professional networking events are some actions that could help create an environment where greater professional growth is possible.

### Contribution to COVID-19 Response

The COVID-19 pandemic devastated many health systems globally. While the availability of vaccinations and enforcement of basic public health precautions are crucially important in reducing morbidity and mortality, having a well-equipped EM workforce on the front line is another important aspect of pandemic management and planning. Of the US-affiliated MEM graduates who participated in this study, 99.4% reported being involved in caring for COVID-19 patients during the pandemic. Almost 40% reported that 76–100% of their work time was spent caring for COVID-19 patients. More than half of graduates were involved in higher level COVID-19 response planning within their clinical department or hospital. The importance of these contributions cannot be understated, particularly considering the devastating impact of the COVID-19 pandemic in India, and the contributions of MEM graduates to the COVID-19 response. Furthermore, our findings highlight the challenges faced by these EPs and exemplify the need for changes to be instituted around the formal recognition of US-affiliated MEM graduates to retain more EM-trained doctors in India.

## LIMITATIONS

This descriptive study focused on the contributions of US-affiliated MEM training program graduates. Therefore, the data is not inclusive of all types of MEM programs in India. Alongside the US-affiliated MEM programs described in this study, other MEM programs have emerged frequently affiliated with local Indian professional societies. We opted to not include non-US affiliated MEM programs in our study. This was a choice we made during the development phase of our project as US-affiliated programs have a more comparable structure, design, and assessment of residents than non-US-affiliated MEM programs.

The survey response rate for this study was 46.9% and may not represent the majority of US-affiliated MEM graduates. The majority of survey respondents also reported graduating within the last five years; thus, older graduates who may be working in other geographic or clinical settings may be under-represented. Nevertheless, our results report the feedback of almost half of all graduates. Given the survey format, this study may have been subject to selection bias, as residents who had a more negative or more positive experience with their MEM education may have felt more or less compelled to complete the survey. The survey was designed and distributed by key US institutional stakeholders in MEM programs, and although no identifying information was collected from respondents, this may have led to bias in the responses. The majority of respondents came from the GW-affiliated MEM programs. This was expected as GW-affiliated MEM programs possess the majority of the Indo-US program sites and seats compared to other US institutions. As a result, this study may overly represent these graduates. Graduates from Northwell Health-affiliated programs had the highest survey response rate compared to other programs, although they only contributed to 9.6% of all survey respondents. Contact information was unavailable for 22 of the 736 (1.7%) MEM graduates; thus, they were not included in the study. This small percentage is likely negligible, and we do not believe it significantly influenced the findings of our study.

## CONCLUSION

The results of this report describe the contributions of US-affiliated Masters in Emergency Medicine graduates on EM specialty development in India. Our survey-based report highlights the important role of MEM graduates over the last 15 years. The role of these graduates should be considered as a means to expedite EM specialty development, systems development, and clinical care across India. As India works to develop more formal training pathways, MEM graduates will continue to contribute to the delivery of patient care and the growth of the emergency care system.

## Supplementary Information



## Figures and Tables

**Table 1:** Demographics of survey participants.

(# of respondents, % of respondents)	
What year did you graduate from your MEM program?	
2020 (60, 17.9%)	
2019 (46, 13.7%)	2013 (13, 3.9%)
2018 (42, 12.5%)	2012 (7, 2.1%)
2017 (42, 12.5%)	2011 (5, 1.5%)
2016 (40, 11.9%)	2010 (3, 0.9%)
2015 (32, 9.6%)	2009 (0, 0.0%)
2021 (27, 8.1%)	2008 (0, 0.0%)
2014 (18, 5.4%)	2007 (0, 0.0%)
Which United States-affiliate is associated with your MEM program?	
George Washington University (300, 89.6%)	
Northwell Health (formerly North Shore – LIJ Health System) (32, 9.6%)	
State University of New York Upstate (2, 0.6%)	
University of Maryland (1, 0.3%)	
Within which hospital did you complete your MEM training?	
Kokilaben Dhirubhai Ambani Hospital (KDAH) (49, 14.6%)	
Peerless (48, 14.3%)	MAX Shalimar Bagh (11, 3.3%)
Aster MIMS Calicut (36, 10.7%)	Believer’s Church (8, 2.4%)
Mission Hospital - Durgapur (32, 9.6%)	Aster CMI Bangalore (6, 1.8%)
MAX SAKET (25, 7.5%)	MAX Smart (6, 1.8%)
Moolchand (18, 5.4%)	Other (4, 1.2%)
Baby Memorial Hospital (16, 4.8%)	MAX Dehradun (3, 0.9%)
MAX PPG (14, 4.2%)	Global Hospital - Chennai (2, 0.6%)
Aster Medcity Kochi (14, 4.2%)	MAX Vaishali (2, 0.6%)
Meenakshi Mission Hospital & Research Center (12, 3.6%)	KIMS - Trivandrum (2, 0.6%)
AMRI - Bhubaneswar (12, 3.6%)	MAX Mohali (1, 0.3%)
Global Hospital - Bangalore (12, 3.6%)	DM Academy Wayanad (1, 0.3%)
	Apollo Glenn Eagles Hospital - Kolkata (1, 0.3%)

*MEM*, Masters in Emergency Medicine.

**Table 2:** Work environment and clinical contribution of Masters in Emergency Medicine graduates.

(# of respondents, % of respondents)	
Where do graduates work?	
Within India (210, 62.7%)	Qatar (8, 2.4%)
United Kingdom (65, 19.4%)	Kuwait (5, 1.5%)
United Arab Emirates (17, 5.1%)	Bahrain (2, 0.6%)
Other (10, 3.0%)	Australia (1, 0.3%)
United States (9, 2.7%)	New Zealand (0, 0.0%)
Saudi Arabia (8, 2.4%)	
Within which Indian states are graduates working?	
Delhi NCR (35, 16.8%)	Uttarakhand (3, 1.4%)
Kerala (31, 14.9%)	Assam (3, 1.4%)
Maharashtra (28, 13.5%)	Chhattisgarh (3, 1.4%)
West Bengal (27, 13.0%)	Nagaland (1, 0.5%)
Karnataka (12, 5.8%)	Meghalaya (1, 0.5%)
Odisha (12, 5.8%)	Manipur (1, 0.5%)
Tamil Nadu (10, 4.8%)	Telangana (1, 0.5%)
Uttar Pradesh (8, 3.8%)	Madhya Pradesh (1, 0.5%)
Jharkhand (7, 3.4%)	Arunachal Pradesh (0, 0.0%)
Bihar (6, 2.9%)	Goa (0, 0.0%)
Andhra Pradesh (6, 2.9%)	Himachal Pradesh (0, 0.0%)
Gujarat (4, 1.9%)	Mizoram (0, 0.0%)
Haryana (4, 1.9%)	Sikkim (0, 0.0%)
Punjab (4, 1.9%)	Tripura (0, 0.0%)
Rajasthan (3, 1.4%)	
Why are some graduates practicing outside of India? Difficulties with formal recognition of your MEM certificate in India (97, 79.5%) Difference in salary (71, 58.2%) Difference in standard of living (58, 47.5%)	When did graduates who are practicing outside of India decide to leave India? After finishing the MEM program (96, 80.0%) While I was enrolled in the MEM program (14, 11.7%) Before enrolling in the MEM program (10, 8.3%)
Working environment in India (46, 37.7%)	
Lack of job opportunities in EM in India (15, 12.3%)	
Relocate to be near family (5, 4.1%)	
Opportunity for professional growth (69, 56.6%)	
Other (4, 3.3%)	

*MEM*, Masters in Emergency Medicine, *NCR*, National Capital Region.

**Table 3:** Academic contribution of Masters in Emergency Medicine graduates.

(# of respondents, % of respondents)	
How much time do graduates report teaching emergency medicine to others? 26–50% (129, 38.5%) 0–25% (112, 33.4%) 51–75% (83, 24.8%) 76–100% (11, 3.3%)	What types of education and training are graduates engaged in? Education and training for nurses (218, 66.5%) Education and training for other physicians (191, 58.2%)
Have graduates attended faculty development or teaching courses? No (203, 60.6%) Yes (132, 39.4%)	Education and training for paramedics (191, 58.2%) Community/layperson education (129, 39.3%) Prehospital care development (113, 34.5%) Education and training for ayurvedic healers (34, 10.4%) Other (18, 5.5%)
Have graduates presented research projects or abstracts at a regional, national, or international conference or meeting? Yes (168, 50.5%) No (165, 49.5%)	Have graduates published an abstract, textbook chapter, or research article in a peer-reviewed medical journal? No (215, 64.2%) Yes (120, 35.8%)
Are graduates members of emergency medicine professional organizations within India? (eg, SEMI, INDUS)? Yes (170, 50.7%) No (165, 49.3%)	Have graduates been a part of organizing an emergency medicine conference (national or international)? No (209, 62.4%) Yes (126, 37.6%)
Have graduates attended or completed training in diversity and inclusion or health equity? No (227, 68.0%) Yes (107, 32.0%)	

*MEM*, Masters in Emergency Medicine; *SEMI*, Society of Emergency Medicine India.

**Table 4:** Involvement in the COVID-19 response.

(# of respondents, % of respondents)	
Did graduates treat COVID-19 patients during the COVID-19 pandemic? Yes (333, 99.4%) No (2, 0.6%)	How much time did graduates report spending caring for COVID-19 patients during the pandemic? 76–100% (131, 39.1%) 51–75% (114, 34.0%) 26–50% (76, 22.7%) 0–25% (14, 4.2%)
Did graduates participate in COVID-19 response planning? Yes, within my department (i.e., emergency department, ICU, etc) (178, 53.1%) Yes, within my hospital (105, 31.3%) No (30, 9.0%) Yes, within my city or region (12, 3.6%) Yes, within my state (10, 3.0%)	

*MEM*, Masters in Emergency Medicine; *ICU*, intensive care unit.

**Table 5:** Satisfaction and confidence in Masters in Emergency Medicine training.

(# of respondents, % of respondents)	
My MEM training has made me confident in my ability to practice emergency medicine. Strongly agree (264, 79.0%) Agree (42, 12.6%) Neutral (9, 2.7%) Disagree (3, 0.9%) Strongly disagree (16, 4.8%)	My MEM training has prepared me well for working in the emergency department. Strongly agree (267, 79.7%) Agree (44, 13.1%) Neutral (5, 1.5%) Disagree (3, 0.9%) Strongly disagree (16, 4.8%)
I am satisfied overall with my MEM training. Strongly agree (218, 65.1%) Agree (78, 23.3%) Neutral (12, 3.6%) Disagree (9, 2.7%) Strongly disagree (18, 5.4%)	

*MEM*, Masters in Emergency Medicine.
