# Supplementary material for: Contribution of 15 Years (2007–2022) of Indo-US Training Partnerships to the Emergency Physician Workforce Capacity in India
Source: West J Emerg Med. 2023 Jul 17;24(4):814–22. doi: 10.5811/westjem.59912 (PMC10393456; doi:10.5811/westjem.59912)
Supplement: Supplementary file 1 [file wjem-24-814-s001.docx]

**APPENDIX A: SURVEY INSTRUMENT**

1. Did you complete a 3-year post-graduate MEM program in partnership with a US institution
   (eg, Northwell Health, George Washington University, University of Maryland, State University of New York Upstate, etc)?
   1. Yes
   2. No
2. Provide the name of the hospital where you completed your MEM training program:
   1. Mission Hospital - Durgapur
   2. Meenakshi Mission Hospital & Research Center
   3. KDAH
   4. AMRI - Bhubaneswar
   5. Aster CMI Bangalore
   6. Peerless
   7. Aster MIMS Calicut
   8. Aster Medcity Kochi
   9. MAX SAKET
   10. MAX PPG
   11. MAX Smart
   12. MAX Dehradun
   13. MAX Mohali
   14. MAX Shalimar Bagh
   15. MAX Vaishali
   16. Moolchand
   17. Believer’s Church
   18. Baby Memorial Hospital
   19. DM Academy Wayanad
   20. Global Hospital – Bangalore
   21. Global Hospital – Chennai
   22. RTIICS
   23. KIMS – Trivandrum
   24. Other (please specify)
3. Provide the name of the main US Emergency Medicine affiliate of your MEM program:
   1. George Washington University
   2. Northwell Health (formerly North Shore – LIJ Health System)
   3. University of Maryland
   4. State University of New York Upstate
   5. Other (please specify)
4. Where do you currently work?
   1. Within India
   2. Australia
   3. New Zealand
   4. Kuwait
   5. Saudi Arabia
   6. United Arab Emirates
   7. United Kingdom
   8. United States
   9. Qatar
   10. Bahrain
   11. Other (please specify)
5. If you are practicing outside of India, when did you decide to leave India?
   1. Before enrolling in the MEM program
   2. While I was enrolled in the MEM program
   3. After finishing the MEM program
6. If you chose to practice outside of India, what factors contributed to that decision? (Select all that apply.)
   1. Difference in salary
   2. Difference in standard of living
   3. Difficulties with formal recognition of your MEM degree in India
   4. Lack of job opportunities in EM in India
   5. Working environment in India
   6. Relocate to be near family
   7. Opportunity for professional growth
   8. Other (please describe)
7. If you are practicing in India, within which state of India do you work?
   1. Andhra Pradesh
   2. Arunachal Pradesh
   3. Assam
   4. Bihar
   5. Chhattisgarh
   6. Delhi NCR
   7. Goa
   8. Gujarat
   9. Haryana
   10. Himachal Pradesh
   11. Jharkhand
   12. Karnataka
   13. Kerala
   14. Madhya Pradesh
   15. Maharashtra
   16. Manipur
   17. Meghalaya
   18. Mizoram
   19. Nagaland
   20. Odisha
   21. Punjab
   22. Rajasthan
   23. Sikkim
   24. Tamil Nadu
   25. Telangana
   26. Tripura
   27. Uttarakhand
   28. Uttar Pradesh
   29. West Bengal
8. If you chose to practice in India, what factors contributed to that decision (select all the apply):
   1. Job opportunities
   2. Family reasons
   3. Opportunities for professional growth
   4. Opportunities to contribute to growth of EM in your area/hospital/community/country
   5. Other
9. What year did you graduate from your MEM program?
   1. 2007
   2. 2008
   3. 2009
   4. 2010
   5. 2011
   6. 2012
   7. 2013
   8. 2014
   9. 2015
   10. 2016
   11. 2017
   12. 2018
   13. 2019
   14. 2020
   15. 2021
10. Which work environment best describes where you spend most of your time working?
    1. Emergency department
    2. Outpatient emergency clinic not attached to a hospital
    3. Intensive care unit
    4. Other (please specify)
11. What percentage of your time do you spend performing clinical work (direct involvement in patient care)?
    1. 0-25%
    2. 26-50%
    3. 51-75%
    4. 76-100%
12. What percentage of your time do you spend performing research?
    1. 0-25%
    2. 26-50%
    3. 51-75%
    4. 76-100%
13. What percentage of your time do you spend performing non-clinical work with no direct involvement in patient care (eg, administration)? This does not include time dedicated to teaching.
    1. 0-25%
    2. 26-50%
    3. 51-75%
    4. 76-100%
14. What percentage of your time do you spend teaching emergency medicine to others?
15. 0-25%
16. 26-50%
17. 51-75%
18. 76-100%
19. Have you attended any faculty development program or teaching courses?
    1. Yes
    2. No
20. Are you teaching emergency medicine to others in a formal postgraduate emergency medicine training program (Masters in Emergency Medicine, MD Emergency Medicine, DNB Emergency Medicine, Fellowship in Emergency Medicine, etc) as part of your work?
    1. Yes
    2. No
21. Have you served as an examiner for MRCEM exams?
    1. Yes
    2. No
22. Have you served as an examiner for MEM exams?
    1. Yes
    2. No
23. Have you attended or completed any training/courses/workshops in inclusion, diversity and equity?
    1. Yes
    2. No
24. If yes, please mention details.
25. Have you presented a research projector abstract at a regional, national, or international meeting or medical conference?
    1. Yes
    2. No
26. Have you published an abstract, textbook chapter, or research article in a peer-reviewed medical journal?
    1. Yes
    2. No
27. Have you given a lecture on an emergency medicine topic at the regional, national, or international level?

a. Yes

b. No

1. Are you involved in any of the following emergency medicine community outreach activities? (Select all that apply.)
   1. Prehospital care development
   2. Community/layperson education
   3. Education and training for paramedics
   4. Education and training for nurses
   5. Education and training for other physicians
   6. Education and training for ayurvedic healers
   7. Other (please describe)
2. Do you belong to an emergency medicine professional organization in India (eg, SEMI, INDUS)?
   1. Yes
   2. No
3. Do you belong to an international emergency medicine association (IFEM,ACEP,ASEM, etc)?
   1. Yes
   2. No
4. Have you completed the MRCEM (Member of the Royal College of Emergency Medicine) UK certification exam?
   1. Yes
   2. No
   3. In the process
5. If yes or in the process, why did you take the MRCEM exam?
   1. Increase professional opportunities within India?
   2. Planning to leave India
   3. Other (please describe)
6. If no, do you plan to take the MRCEM certification exam within the next 3 years?
   1. Yes
   2. No
7. If yes, why?
   1. Increase professional opportunities within India?
   2. Planning to leave India
   3. Other (please describe)
8. Are you considering pursuing emergency medicine subspecialty training, such as in ultrasound, pediatrics, administration, etc?
   1. Yes, I am considering this.
   2. No, I am not considering this.
   3. I have already received subspecialty training in [*free text field*)
9. Have you been a part of organizing an emergency medicine conference (national, international)
   1. Yes
   2. No
10. I feel confident that I could get a job in emergency medicine anywhere in India with my MEM training.

(Strongly Disagree) 1 – 2 – 3 – 4 – 5 (Strongly Agree)

1. I feel confident that I could get a job in emergency medicine anywhere outside of India with my MEM training.

(Strongly Disagree) 1 – 2 – 3 – 4 – 5 (Strongly Agree)

1. I feel that my MEM training has made me confident in my ability to practice emergency medicine.

(Strongly Disagree) 1 – 2 – 3 – 4 – 5 (Strongly Agree)

1. My MEM training program prepared me well for working in the emergency department.

(Strongly Disagree) 1 – 2 – 3 – 4 – 5 (Strongly Agree)

1. I am satisfied overall with my MEM training.

(Strongly Disagree) 1 – 2 – 3 – 4 – 5 (Strongly Agree)

1. Did you treat COVID-19 patients during the COVID-19 pandemic?
   1. Yes
   2. No
2. What percentage of your clinical time did you spend caring for COVID-19 patients during the pandemic?
   1. 0-25%
   2. 26-50%
   3. 51-75%
   4. 76-100%
3. Did you participate in COVID-19 response planning?
   1. No
   2. Yes, within my department (eg, emergency department, ICU, etc)
   3. Yes, within my hospital
   4. Yes, within my city or region
   5. Yes, within my state
